# Visualization and quantification of the cellular and extracellular components of *Salmonella* Agona biofilms at different stages of development

**DOI:** 10.1371/journal.pone.0200011

**Published:** 2018-07-10

**Authors:** Camino González-Machado, Rosa Capita, Félix Riesco-Peláez, Carlos Alonso-Calleja

**Affiliations:** 1 Department of Food Hygiene and Technology, University of León, León, Spain; 2 Institute of Food Science and Technology, University of León, León, Spain; 3 Department of Electrical Engineering and Systems Engineering and Automatic Control, University of León, León, Spain; Ross University School of Medicine, DOMINICA

## Abstract

*Salmonella* is a major food-borne pathogen able to persist in food processing environments because of its ability to form biofilms. A *Salmonella enterica* serotype Agona isolate from poultry (S24) was grown at 37°C in biofilms for up to 144 hours (H144) in attachment to polystyrene surfaces. Biofilm structures were examined at different stages in their development (H3, H24, H48, H72, H96 and H144) using confocal laser scanning microscopy (CLSM) in conjunction with fluorescent dyes for live cells (SYTO 9), dead cells (propidium iodide), proteins (fluorescein isothiocyanate isomer I), lipids (DiD’oil), α-polysaccharides (concanavalin A, tetramethylrhodamine conjugate), and β-polysaccharides (calcofluor white M2R). Strain S24 developed a robust biofilm at H72 (biovolume of 166,852.5 ± 13,681.8 μm^3^ in the observation field of 16,078.2 μm^2^). The largest biovolume of live cells was also detected at H72 (128,110.3 ± 4,969.1 μm^3^), decreasing thereafter, which was probably owing to the detachment of cells prior to a new phase of colonization. The percentage of dead cells with regard to total cells in the biofilms increased throughout the incubation, ranging from 2.3 ± 1.1% (H24) to 44.2 ± 11.0% (H144). Proteins showed the greatest biovolume among the extracellular components within the biofilms, with values ranging from 1,295.1 ± 1,294.9 μm^3^ (H3) to 19,186.2 ± 8,536.0 μm^3^ (H96). Maximum biovolume values of 15,171.9 ± 660.7 μm^3^ (H48), 7,055.3 ± 4,415.2 μm^3^ (H144), and 2,548.6 ± 1,597.5 μm^3^ (H72) were observed for β-polysaccharides, α-polysaccharides and lipids, respectively. A strong (*P* < 0.01) positive correlation was found between the total biovolume of biofilm and the biovolume of live cells, proteins and β-polysaccharides, which may serve as useful markers of biofilm formation. The present work provides new insights into the formation of *S*. Agona biofilms. Our findings may contribute to the designing of reliable strategies for preventing and removing these bacterial communities.

## Introduction

*Salmonella* is a globally widespread food-borne pathogen. In the European Union, this bacterium has become the most frequently reported cause of outbreaks of food-borne illness, resulting in a total of 94,530 confirmed cases of human salmonellosis in 2016, with an incidence rate of 20.4 cases per 100,000 population. *Salmonella enterica* serotype Agona was among the fifteen most common serotypes in human cases arising in the European Union during 2016 [1].

*Salmonella* has the ability to form biofilms, which have been identified as an important factor for the persistence of food-borne pathogens in food-processing environments [2, 3, 4]. *Salmonella* Agona strains have been classified as strong biofilm producers [5]. Biofilms have been shown to be the main source of the contamination of foodstuffs and have been associated with many outbreaks of food-borne illness, becoming a significant problem in the food industry [6]. Serious engineering problems are also associated with the presence of biofilms on the equipment and in the installations of food-processing facilities [5]. Additionally, there is a correlation between biofilm formation and the ability of strains to colonize and replicate within the intestines of multiple host species, including humans [7].

Biofilms are structured microbial communities enclosed in a self-produced matrix of hydrated extracellular polymeric substances (EPS) and are adhered to an inert or living surface [8]. The EPS, which is composed of a wide variety of organic materials, including alfa-polysaccharides (e.g. α-mannopyranosyl and α-glucopyranosyl residues), beta-polysaccharides (e.g. cellulose), proteins (e.g. flagella and curli fimbriae), nucleic acids and lipids [9, 10], is thought to maintain the biofilm architecture and functions, holding biofilm cells together and protecting them from environmental stresses. On these lines, it has been reported that cells in biofilms are far less sensitive to sanitizing agents than their non-attached individual planktonic counterparts [11].

In view of the crucial role that the EPS matrix plays in biofilm development, resistance to chemicals and detachment [12, 13], a thorough understanding of the extracellular matrix ultrastructure is critical for the rational design of strategies aimed at disrupting biofilms. This would help in preventing the numerous critical problems in terms of public health and financial losses that are associated with these structures in both industrial and clinical settings.

Confocal laser scanning microscopy (CLSM) is among the most versatile and effective approaches for understanding the architecture of biofilms and their development over time. When CLSM is combined with a range of fluorescent probes, a three-dimensional image of various cellular and extracellular biofilm constituents can be obtained together with detailed quantitative information about them [14]. Thus, the quantitative three-dimensional results recorded by CLSM are an important basis for understanding, controlling and modelling of biofilms formed by pathogenic bacteria. In previous work, the structural parameters and cellular viability of 48-hour-old *Salmonella* Typhimurium biofilms were studied using CLSM [2]. However, it would appear that the individual cellular and EPS matrix components of biofilms formed by *Salmonella* at different stages of development have not hitherto been visualized or quantitatively characterized.

Taking into account the significant impact of *Salmonella* biofilms in the food industry, studies require to be conducted to elucidate the dynamics of the various different components of these structures during the biofilm formation process. This would facilitate progress toward the goal of developing more effective specific methods to control these bacterial communities. Therefore, the aim of this study was to visualize and characterize quantitatively, through CLSM together with image analysis techniques, the main cellular and extracellular components of biofilms formed at 37°C on polystyrene surfaces by a *Salmonella* Agona strain of food origin over the course of 144 hours. This would appear to be the first time that a quantitative analysis of live cells, dead cells, proteins, lipids, α-polysaccharides and β-polysaccharides content in the *S*. Agona biofilm at different stages of development has been performed.

## Materials and methods

### Bacterial strain and culture conditions

A *Salmonella enterica* serotype Agona strain (S24) available in our laboratory, previously isolated from a chicken carcass in a local slaughterhouse, was used. The strain was preserved in tryptone soya broth (TSB, Oxoid Ltd., Hampshire, England) supplemented with 20% (v/v) glycerol at -80°C. Prior to the experiments, the frozen cells were sub-cultured twice in TSB at 37°C. Working cultures were kept at 4°C on plates of tryptone soya agar (TSA, Oxoid).

### Biofilm formation

S24 cultures were grown in TSB for 24 hours at 37°C, and appropriate (two-fold) dilutions in the same culture broth were made to obtain a concentration of approximately 10^7^ cfu/ml. A volume of 250 μl of this culture was added to the wells of Nunc™ MicroWell™ 96-Well Optical-Bottom Plates with Polymer Base (Thermo Fisher Scientific, New Hampshire; reference number 165305). These are of high optical quality, and have a low fluorescent background and overall flatness, which allowing high resolution imaging.

After one hour of adhesion at 37°C, the wells were rinsed with 150 mM of NaCl in order to eliminate any non-adherent bacteria before being refilled with TSB. The plates were then incubated for 3, 24, 48, 72, 96 or 144 hours at 37°C. After incubation, the wells were rinsed with 150 mM of NaCl.

### Staining procedure

Six fluorescent dyes were used (Table 1). SYTO 9 and propidium iodide (PI) from the LIVE/DEAD^®^ BacLight^TM^ Bacterial Viability Kit, DiIC_18_(5) oil, 1,1'-dioctadecyl-3,3,3',3'-tetramethylindodicarbocyanine perchlorate (DiD’oil) and concanavalin A, tetramethylrhodamine conjugate (ConA-TMR) were purchased from Invitrogen (Carlsbad, California), while fluorescein isothiocyanate isomer I (FITC) and calcofluor white M2R (CFW) were purchased from Sigma (St Louis, Missouri).

**Table 1:** Stains and microscopic parameters used in study the cellular and extracellular components of the biofilms formed by *Salmonella* Agona.

Dye	SYTO 9	PI^1^	FITC^2^	DiD’oil^3^	ConA-TMR^4^	CFW^5^
Pinhole	44 μm	44 μm	50 μm	56 μm	52 μm	40 μm
Laser Wavelength	488 nm: 0,20%	561 nm: 4,50%	488 nm: 0,20%	640 nm: 2,00%	561 nm: 4,50%	405 nm: 3,00%
Excitation Wavelength	483 nm	305 nm	495 nm	648 nm	552 nm	254 nm
Emission Wavelength	500 nm	617 nm	519 nm	670 nm	578 nm	432 nm
Detection Wavelength	450–560 nm	560–700 nm	400–700 nm	645–700 nm	400–700 nm	400–560 nm
Imaging Device	LSM 800 / Airyscan	LSM 800 / GaAsP-Pmt2	LSM 800 / GaAsP-Pmt1	LSM 800 / GaAsP-Pmt2	LSM 800 / GaAsP-Pmt1	LSM 800 / GaAsP-Pmt1
Detector	Airyscan	GaAsP	GaAsP	GaAsP	GaAsP	GaAsP
Detector Gain	700 V	660 V	700 V	800 V	660 V	850 V
**Target**	**Live cells**	**Dead cells**	**Proteins**	**Lipids**	**α-polysaccharides**	**β-polysaccharides**

^1^, propidium iodide

^2^, fluorescein isothiocyanate isomer I

^3^, DiIC_18_(5) oil, 1,1'-dioctadecyl-3,3,3',3'-tetramethylindodicarbocyanine perchlorate

^4^, concanavalin A, tetramethylrhodamine conjugate

^5^, calcofluor white M2R.

Five working solutions of stains were prepared in NaCl 150 mM: SYTO 9 (stock 3.34 mM in DMSO) plus PI (stock 20 mM in DMSO) at 1.0 μl/ml each (A), FITC (stock 2 mg in 100 μl absolute ethanol) at 46.6 μg/ml (B), DiD’oil (stock of 25 mg in 2.5 ml of absolute ethanol) at 79.4 μg/ml (C), ConA-TMR (10 mg in 2 ml distilled water with 16,8 mg NaHCO_3_) at 944.8 μg/ml (D) and CFW at 189 μl/ml (E). To avoid overlapping spectra, a volume of 250 μl of each of the five solutions was added to five different wells (A to E, respectively). Additionally, in order to calculate the structural parameters of the complete biofilm (all components simultaneously), a sixth well (F) was refilled with 250 μl of NaCl 150 mM including all stains at the above-mentioned concentrations. The microtiter plate was then incubated in the dark at 37°C. After 25 minutes wells B, C, D, E and F were rinsed with NaCl at 150 mM and refilled with 250 μl of this saline solution.

### CLSM and image analysis

Confocal laser scanning microscopy (CLSM) image acquisition was performed using a Zeiss LSM 800 Airyscan confocal laser scanning microscope with ZEN 2.3 software (Carl Zeiss, Jena, Germany). Channel mode visualization was done using the 63× (0.8 NA) objective with oil immersion.

Three stacks of horizontal plane images (512 × 512 pixels corresponding to 126.8 × 126.8 μm) with a z-step of 0.25 μm, were acquired for each well from three different randomly chosen areas. Two independent experiments were performed for each development stage. Thus, a total of 216 CLSM images were obtained (6 development stages by 2 replicates by 6 wells by 3 surfaces in each well). For image analysis, original Zeiss files (CZI format) were imported into the IMARIS 9.1 software package (Bitplane, Zurich, Switzerland) for modelling in three dimensions.

The quantitative structural parameters of the biofilms were calculated using the BioRCA 1.7 software, previously developed by some members of our Research Group using the *Lazarus* Integrated Development Environment (IDE). From a threshold value, each pixel is labeled as empty or marked. With this information, a three-dimensional model of the biofilm under study is generated and the structural parameters are calculated. This computer program allowed quantification of the total biofilm biomass (comprising all cellular and extracellular components of biofilms) as well as of the individual components of biofilm, represented by fluorescence emitted by SYTO 9 (from cells with intact membranes), PI (from bacteria with damaged membranes), FITC (proteins), DiD’oil (lipids), ConA-TMR (α-polysaccharides) and CFW (β-polysaccharides). Biovolume represented the amount of biofilm (μm^3^) in the observation field of 16,078.2 μm^2^. Surface coverage (%) reflected the efficiency of substratum colonization by the populations of bacteria. Roughness provided a measure of how much the thickness of the biofilm varied and was thus an indicator of biofilm heterogeneity [15]. A roughness with a value of zero indicates a biofilm of uniform thickness, and the greater the roughness coefficient, the rougher the surface. The maximum thickness (μm) of biofilms was determined directly from the confocal stack images.

### Statistical analysis

The quantitative structural parameters of the biofilms were compared for statistical significance using analysis of variance techniques (ANOVA) and Duncan's multiple range test. A correlation analysis was performed to determine the relationship between the different structural parameters and components of the biofilms. Data processing was performed using the Statistica® 8.0 software package (StatSoft Ltd., Tulsa, Oklahoma).

## Results

### Structural parameters of the *Salmonella* Agona biofilms

The structural parameters of the *S*. Agona biofilms at different stages of development were evaluated by CLSM observations and digital image analysis. As expected, the lowest total biomass of biofilms was observed at H3 (6,600.7 ± 1,245.6 μm^3^ in the observation field of 16,078.2 μm^2^), as shown in Table 2. Biovolume increased strongly up to H72 (166,852.5 ± 13,681.8 μm^3^) and decreased thereafter. Biofilm expansion during the period from H3 to H72 was multidirectional, in both horizontal (increase in surface coverage) and vertical (increase in thickness) directions. After 48 hours, biofilms covered most of the surface available. On the other hand, a thin biofilm (from 0.44 ± 0.04 μm to 2.68 ± 0.15 μm, as average) was observed up to H48, with growth in depth becoming significant after this point. The greatest thickness was reached at H72, 10.17 ± 1.89 μm being the average thickness and 32.10 ± 2.14 μm the maximum. Roughness values decreased from H3 to H72 and increased thereafter. A positive change in the roughness coefficient indicated an increase in biofilm heterogeneity.

**Table 2:** Structural parameters of the biofilms formed by *Salmonella* Agona on polystyrene after varying periods of development.

Incubation time (hours)	Biovolume (μm^3^)	Surface coverage (%)	Thickness (μm)		Roughness
			Maximum	Average	
H3	6,600.7 ± 1,245.6a	38.32 ± 3.36a	9.07 ± 1.05a	0.44 ± 0.04a	1.29 ± 0.04a
H24	27,446.3 ± 4,561.9b	89.27 ± 1.06b	14.47 ± 0.67b	1.52 ± 0.08ab	0.78 ± 0.07b
H48	51,726.5 ± 5,011.2c	98.83 ± 1.78c	20.96 ± 3.37c	2.68 ± 0.15b	0.48 ± 0.06c
H72	166,852.5 ± 13,681.8d	99.87 ± 0.06c	32.10 ± 2.14d	10.17 ± 1.89c	0.32 ± 0.03d
H96	94,616.1 ± 14,232.7e	98.76 ± 1.18c	21.40 ± 2.70c	6.97 ± 0.45d	0.52 ± 0.10c
H144	91,143.9 ± 12,519.4e	87.90 ± 4.97b	33.27 ± 0.68d	6.36 ± 0.71d	0.82 ± 0.03b

Data (mean ± SD) in the same column with no letters in common are significantly different (*P* < 0.05).

A positive correlation (from 0.630 to 0.941; *P* < 0.01) was found between biovolume, surface coverage, maximum thickness and average thickness of biofilms. All of these were negatively correlated with biofilm roughness (from -0.915 to -0.609; *P*<0.01), as shown in Table 3.

**Table 3:** Coefficients of correlation between various structural parameters of the biofilms formed by *Salmonella* Agona on polystyrene over the course of 144 hours of incubation.

		Total biofilm					Individual components within biofilm					
		Biovolume	Surface coverage	Maximum thickness	Average thickness	Roughness	Live cells	Dead cells	Proteins	Lipids	α-polysac.	β-polysac.
Total biofilm												
	Biovolume	1.000										
	Surface coverage	0.630**	1.000									
	Maximum thickness	0.829***	0.632**	1.000								
	Average thickness	0.941***	0.609**	0.845***	1.000							
	Roughness	-0.693**	-0.915***	-0.609**	-0.689**	1.000						
Individual components within biofilm											
	Live cells	0.941***	0.513*	0.684**	0.876***	-0.646**	1.000					
	Dead cells	0.540*	0.322	0.627**	0.522*	-0.167	0.269	1.000				
	Proteins	0.778***	0.710***	0.708**	0.735***	-0.659**	0.597**	0.532*	1.000			
	Lipids	0.467	0.457	0.332	0.519*	-0.534*	0.536*	-0.078	0.408	1.000		
	α-polysaccharides	0.341	0.200	0.585*	0.346	-0.079	0.068	0.752***	0.532*	-0.222	1.000	
	β-polysaccharides	0.685**	0.797***	0.717***	0.665**	-0.850***	0.549*	0.369	0.696**	0.272	0.271	1.000

***, *P* < 0.001

**, *P* < 0.01

*, *P* < 0.05.

### Cellular and extracellular components within the *Salmonella* Agona biofilm

**Fig 1:**
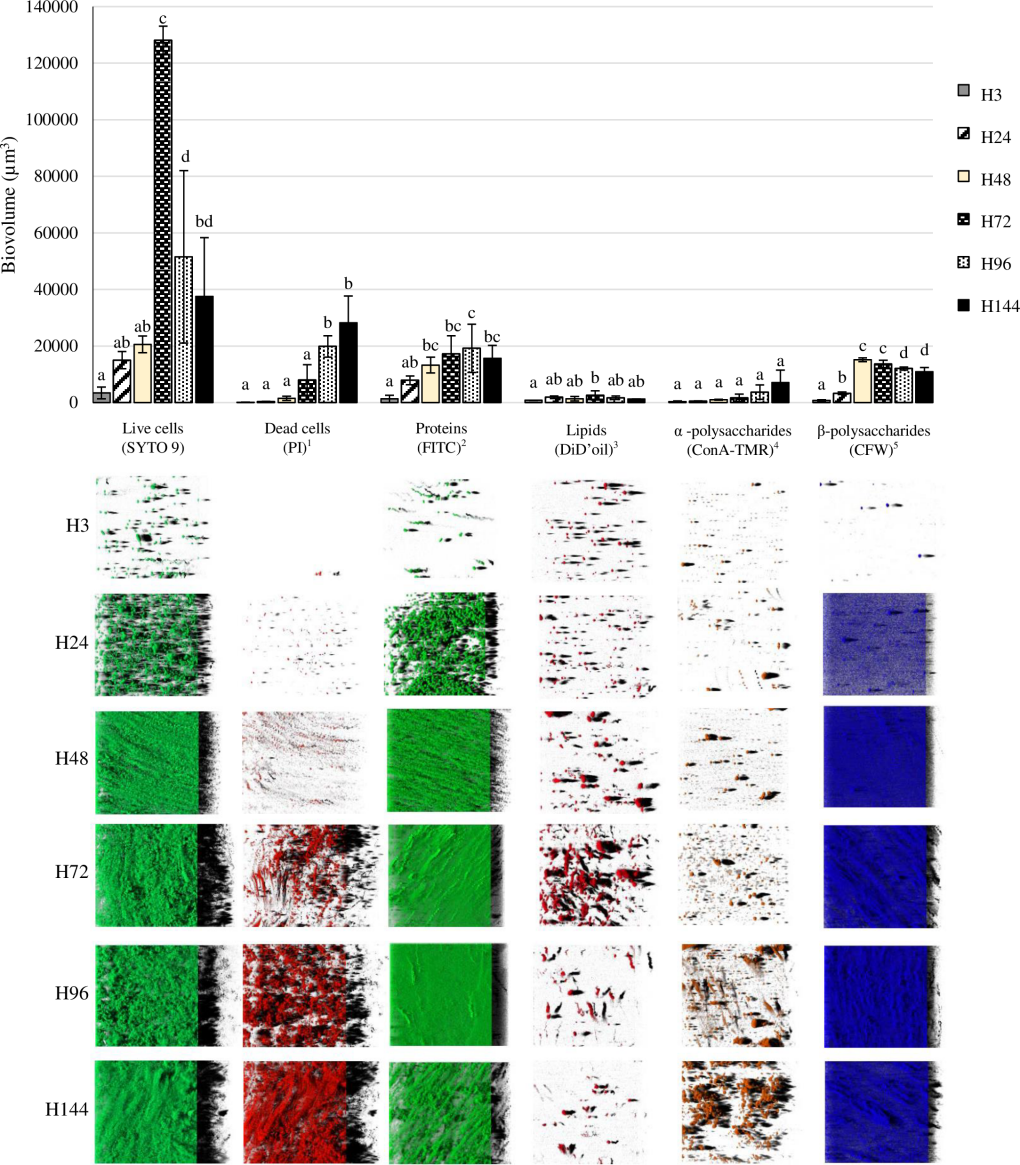
Biovolume of the individual components in biofilms formed by *Salmonella* Agona on polystyrene after varying periods of development. ^1^, propidium iodide; ^2^, fluorescein isothiocyanate isomer I; ^3^, DiIC_18_(5) oil, 1,1'-dioctadecyl-3,3,3',3'-tetramethylindodicarbocyanine perchlorate; ^4^, concanavalin A, tetramethylrhodamine conjugate; ^5^, calcofluor white M2R. Bars (mean ± SD) in the graph for the same fluorescent dye without any letter in common are significantly different (*P* < 0.05). The images correspond to three-dimensional reconstructions obtained from confocal stack images by the IMARIS 9.1 software, virtual projections being included on the right.

Fig 1 shows biomass measurements of each of the biofilm components after 3, 24, 48, 72, 96 and 144 hours of incubation. Representative three-dimensional renderings of individual components of the *S*. Agona biofilms are also shown.

Initially (H3) only single *S*. Agona live cells (SYTO 9-stained) and small clusters consisting of a few live bacteria were seem by CLSM. After 24 hours of incubation, the quantity of sessile cells had increased, and irregularly shaped micro-colonies were observed. As the incubation time increased, the images of live cells changed from micro-colonies to compact structures growing up until H72. Moreover, the contact surfaces were completely covered by dense and homogeneous biofilm of live cells when cultured for 72 hours. At this point the strain exhibited the greatest biofilm thickness under these conditions (Fig 1). Thus, the largest biovolume of live cells (128,110.3 ± 4,969.1 μm^3^) and the highest percentage of biovolume of live cells within the total biovolume of the biofilm (74.9 ± 2.5%) was seen at H72. A decline in the biovolume and percentage of live cells was observed after this point, with the biofilm becoming thinner and more wrinkled.

**Fig 2:**
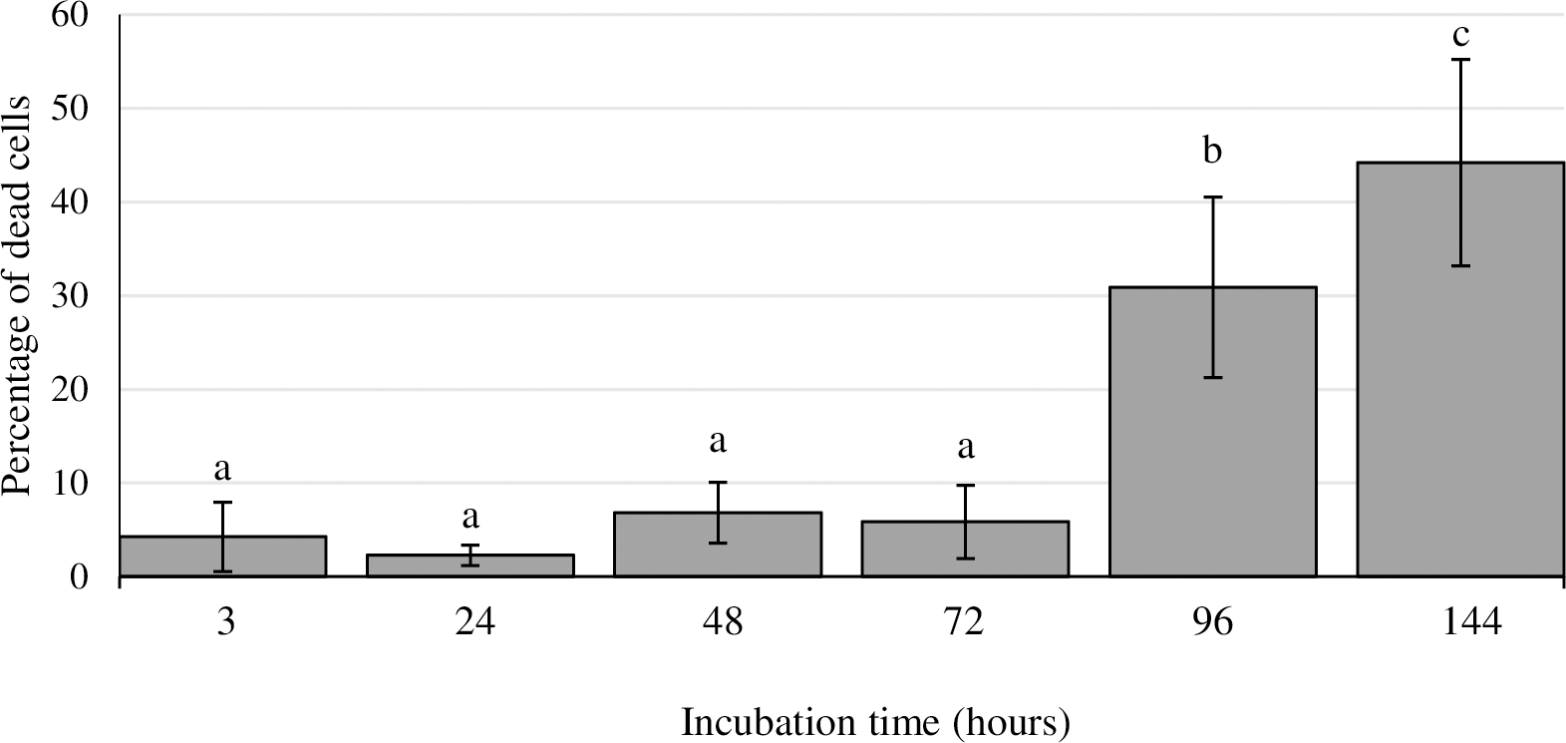
Percentage of dead cells relative to total cells in biofilms formed by *Salmonella* Agona on polystyrene after varying periods of development. Bars (mean ± SD) with no letters in common are significantly different (*P* < 0.05).

Staining the biofilm with propidium iodide (PI) showed the presence of red aggregates of materials probably formed by a mixture of dead or damaged cells and extracellular DNA (eDNA). These increased significantly over the course of storage, from 123.2 ± 70.2 μm^3^ at H3 to 28,176.5 ± 9,509.6 μm^3^ at H144 (Fig 1). The percentage of dead cells (PI-stained) relative to total cells ranged from 2.3 ± 1.1% at H24 to 44.2 ± 11.0% at H144 (Fig 2).

The individual components within the EPS matrix of *S*. Agona biofilms at various stages of development was also monitored. Proteins and β-polysaccharides (FITC- and CFW-stained compounds, respectively) were major components of the biofilm matrix. The biovolume of proteins in the observation field ranged from 1,295.1 ± 1,294.9 μm^3^ at H3 to 19,186.2 ± 8,536.0 μm^3^ at H96, which may be seen from Fig 1. A low biovolume of β-polysaccharides was observed after 3 hours of incubation (761.7 ± 256.7 μm^3^). Quantification of this biofilm component showed a progressive accumulation of biomass during the first 48 hours, the point at which the highest biovolume was observed (15,171.9 ± 660.7 μm^3^). On the other hand, BioRCA analysis of CLSM images revealed low biovolumes for fluorescent labelled lipids (stained with DiD’oil, these lying between 799.6 ± 26.4 μm^3^ at H3 and 2,548.6 ± 1,597.5 μm^3^ at H72) and α-polysaccharides (ConA-TMR; ranging between 306.8 ± 255.7 μm^3^ at H3 and 7,055.3 ± 4,415.2 μm^3^ at H144) (Fig 1).

The biovolume of proteins and β-polysaccharides increased in a similar way to the total biovolume of the biofilms. Significant positive correlations (from 0.685 to 0.778; *P* < 0.01) were found between these three structural parameters. On the other hand, no significant correlations were observed between the biovolume of lipids, the biovolume of α-polysaccharides and the total biovolume of biofilm (Table 3).

**Fig 3:**
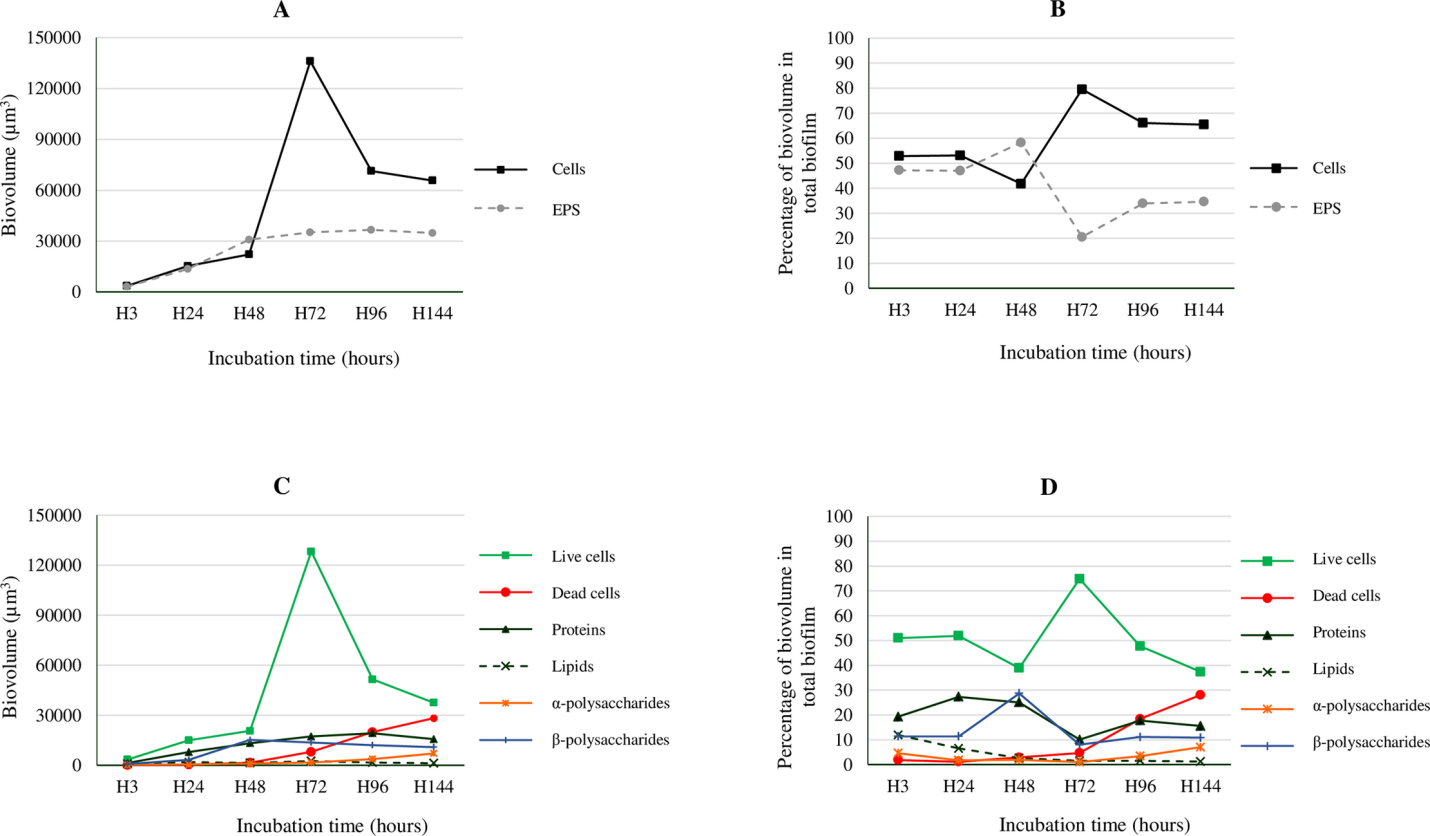
Biovolumes of cells and EPS in biofilms formed by *Salmonella* Agona on polystyrene over the course of 144 hours of incubation at 37°C. A, cells and extracellular polymeric substances (EPS), expressed as the biovolume (μm^3^) in the observation field (16,078.2 μm^2^); B, cells and EPS, expressed as the percentage of biovolume relative to total biovolume of the biofilm; C, live cells, dead cells, proteins, lipids, α-polysaccharides and β-polysaccharides, expressed as the biovolume (μm^3^) in the observation field (16,078.2 μm^2^); D, live cells, dead cells, proteins, lipids, α-polysaccharides and β-polysaccharides, expressed as the percentage of biovolume relative to the total biovolume of the biofilm.

Cellular biomass (the sum of components stained with SYTO 9 and PI) rose from 3,543.6 ± 2,091.5 μm^3^ at H3 to 22,097.1 ± 3,014.7 μm^3^ at H48. The biomass of cells rose markedly from H48 onwards, reaching its highest value at H72 (136,104.2 ± 2,604.3 μm^3^), but decreased afterwards. The biovolume of the EPS matrix varied between 3,163.2 ± 1,747.5 μm^3^ at H3 and 36,590.7 ± 10,038.3 μm^3^ at H96 (Fig 3A). Because similar (*P* > 0.05) biovolumes of EPS were observed at H48 (30,804.0 ± 3,351.4 μm^3^) and H72 (35,138.6 ± 6,902.6 μm^3^), the variation in the biovolume of cells in the biofilm seems to be responsible for the marked decrease (*P* < 0.05) in the percentage of EPS between H48 (58.3 ± 2.1%) and H72 (20.4 ± 3.0%), visible in Fig 3B. The ratio between cells and EPS was observed to range from 0.72 ± 0.06 at H48 to 3.96 ± 0.67 at H72.

The formation of micro-colonies and the EPS matrix went through three distinct developmental phases during the formation of biofilm. The early phase (H3 to H24) was characterized by the attachment of a number of bacterial cells to the polystyrene surface, forming small clusters and micro-colonies, and by the start of the formation of the EPS matrix. Both cellular and EPS biomass showed a substantial increase from H3 to H24. During this phase, similar values were observed for the biovolume of cells and of EPS (the total of the extracellular components measured), as may be observed from Fig 3A.

In the second phase (H24 to H72) a compact biofilm was formed, resulting in a marked increase in its cellular biomass. During this period, the biovolume of EPS and their individual components did not show any significant changes (Fig 3A and 3C). After 72 hours of biofilm formation, a third phase saw an increase in the biovolume of dead cells, as well as a marked decrease in the biovolume of live cells (probably as a consequence of biofilm dispersal) (Fig 3C). Biovolumes of individual cellular and EPS matrix components, expressed as percentage of total biovolume of the biofilm, are shown in Fig 3D.

## Discussion

### Structural parameters of the *Salmonella* Agona biofilms

In the food industry, biofilms formed by food-borne pathogens have become a major public health issue and a great financial concern. This is because of the potential contamination of foodstuffs and the greater resistance to disinfectants of biofilm relative to planktonic forms. Indeed, biofilm-related contamination of equipment has been estimated to cause nearly 60% of outbreaks of food-borne disease [16]. A better understanding of the formation and ultrastructure of biofilms may open up avenues for the control of these structures. The work reported here would appear to be the first presentation of a numerical characterization over time of the biofilms formed on polystyrene by a *S*. Agona strain of food origin. In this study, *Salmonella* biofilms were studied at 37°C in order to test the behaviour of this pathogen at extreme room temperatures, and because this is the optimal growth temperature for *Salmonella* and the human body temperature.

*Salmonella* biofilms quickly and easily established themselves on polystyrene surfaces by building a three-dimensional structure with multiple layers of cells. This finding is a matter for concern because plastic materials are frequently used in the installations and equipment of food-processing facilities [5]. It should be noted that certain of the results of the research being reported here show substantial standard deviations. This may be explained by taking into account the heterogeneity of the biofilms, possibly due to the presence of clusters and deeper emptier areas.

The results, as shown in Table 2, are in agreement with the findings of Bridier et al. [17], who demonstrated that *S*. *enterica* strains of different serotypes (including a *S*. Agona isolate) produced biofilms on hydrophobic (polystyrene) surfaces that had biovolume values of approximately 100,000 μm^3^ in the observation field of 14,209 μm^2^, with a maximum thickness of 35 μm, and a roughness of 0.4. Structural parameter values in the present work are also similar to those previously observed for biofilms formed on polystyrene by a *S*. Typhimurium isolate from food (S175), as recorded by Capita et al. [2]. It should be noted that in both the above-mentioned papers only fluorescent dyes for staining nucleic acids were used, and the EPS matrix was not studied.

### Cellular and extracellular components within the *Salmonella* Agona biofilm

Biovolume and percentage of live cells within the biofilm decreased after 72 hours of incubation. This decrease, which has previously been observed for several groups of food-borne pathogenic bacteria, is likely to be due to the detachment of cells prior to a new phase of colonization [18, 19]. The increase in the biovolume of dead or damaged cells could also contribute to the decrease of percentage of live cells from H72.

The presence of eDNA in *Salmonella* biofilms is in accordance with previously published reports [20]. The origin of the eDNA composing the biofilms remains unclear but might involve cellular lysis as a consequence of the biofilm life cycle or the release of small vesicles, as has formerly been put forward [21, 22]. A key role of eDNA from lysed cells in the intercellular adhesion and stability of biofilm structure has also been suggested [20].

The major part played by the EPS matrix in biofilms includes the adhesion and aggregation of cells, their cohesion, the retention of water, the sorption of organic and inorganic material, enzymatic activity, acting as a nutrient source and exchange of genetic information, the export of cell components and cell-to-cell communication [23]. Moreover, the EPS matrix increases resistance to antimicrobial agents, in comparison with that of more vulnerable planktonic cells. Thus, the control of biofilms does not necessarily require the direct killing of the bacteria in the biofilm but might be directed toward the degradation or dispersal of the EPS matrix. This would reverse the sessile mode of growth to a planktonic state, which is significantly easier to remove or delete, for example by the use of disinfectants. In such a scenario, a better understanding of the organization and development of the EPS matrix within biofilms formed by pathogenic bacteria is essential for devising effective strategies for the control and eradication of biofilms. However, there is a lack of studies focusing on individual components of the EPS matrix of *Salmonella* biofilms at different stages of development. In this study, a quantitative characterization of fluorescently labelled structural components of the EPS in biofilms was performed. CLSM combined with image analysis techniques allowed the determination of the predominant matrix components and their spatial distribution at different time points during the formation of biofilm.

The composition of the EPS matrix within *Salmonella* biofilms has been demonstrated to be highly variable, depending upon the environment and the substratum upon which they are formed [10]. In the present study, proteins and β-polysaccharides, in this order, were the predominant components inside the EPS matrix of *S*. Agona biofilms throughout the course of incubation, occurring at a significantly greater volume than lipids and α-polysaccharides. Other authors have also shown proteinaceous components, such as flagella, and polysaccharides to be the key components of the biofilm matrix [6, 10, 20, 23, 24, 25, 26]. As indicated by the positive correlations found, the greater the biovolume of biofilms, the more abundant were components stained with FITC, the dye for proteins, and with CFW, that for β-polysaccharides.

It has been suggested that proteinaceous components provide structural integrity, while polysaccharide components function in a chemically or immune-protective manner or both. It has also been put forward that structural integrity requires complex interactions between both polysaccharides and proteins [10]. According to Arciola et al. [27], polysaccharides are associated with the retention of water, sorption of organic and inorganic compounds, protection against biocides and cohesion. On these lines, Houari et al. [28] demonstrated that an increase in the proportion of polysaccharides (i.e. rendering the matrix dense) ensures better cohesion. Some authors have even suggested that the consistent predominance of polysaccharides reported in the literature for biofilms formed under different growing conditions indicates that carbohydrates may play a greater role in the structural stability of biofilms than do proteins [29].

In agreement with the study being presented here, similar biomass values for cells and for the EPS matrix have also been observed by other authors [9] in *Salmonella* biofilms after a few days of incubation. On the other hand, these results do not agree with most studies, which identified EPS as the main component in mature bacterial biofilms, comprising up to 90% of the biofilm mass in different microbial groups [20, 23]. According to several authors [24, 30], the dominance of EPS over cells might be due to the need for greater protection of the cells from a challenging environment (such as the presence of disinfectants), a protection which was unnecessary under the favourable conditions tested in our study. The variations in biofilm composition observed between different reports can also be due to the use of diverse microbial groups and environmental conditions, such as the temperature at which biofilms were grown. It has been found that the genes involved in curli fimbriae and cellulose production (two important EPS matrix components) by *Salmonella enterica* strains are highly induced when biofilm is formed at temperatures below 30°C [31].

## Conclusions

A better understanding of the biofilms formed by pathogenic bacteria would lead to the design of more efficient control strategies. The present work appears to be the first to present the visualization and quantitative characterization of individual components of the biofilms formed on polystyrene by a *S*. Agona strain of food origin during the course of 144 hours of incubation. This study demonstrates that the EPS matrix of *S*. Agona biofilms contains mainly proteins and β-polysaccharides, which contribute to the biofilm architecture and may be used to identify biofilm formation. The findings of this study may help in determining the physiological state of the biofilms and in identifying and attacking targets so as to prevent and remove these bacterial communities in food industry establishments.

## References

[1] EFSA (European Food Safety Authority) and ECDC (European Centre for Disease Prevention and Control). The European Union summary report on trends and sources of zoonoses, zoonotic agents and food-borne outbreaks in 2016. EFSA J. 2017;15: 5077, 228 pp. https://doi.org/10.2903/j.efsa.2017.5077. 10.2903/j.efsa.2017.5077 PMC7009962 32625371

[2] Capita R, Buzón-Durán L, Riesco-Peláez F, Alonso-Calleja C. Effect of sub-lethal concentrations of biocides on the structural parameters and viability of the biofilms formed by *Salmonella* Typhimurium. Foodborne Pathog Dis. 2017;14(6): 350–356. doi: 10.1089/fpd.2016.2241 28605289 10.1089/fpd.2016.2241

[3] Vestby LK, Møretrø T, Langsrud S, Heir E, Nesse LL. Biofilm forming abilities of *Salmonella* are correlated with persistence in fish meal- and feed factories. BMC Vet Res. 2009;5: 20 doi: 10.1186/1746-6148-5-20 19473515 10.1186/1746-6148-5-20 PMC2693496

[4] Vogeleer P, Tremblay YDN, Mafu AA, Jacques M, Harel J. Life on the outside: role of biofilms in environmental persistence of Shiga-toxin producing *Escherichia coli*. Front Microbiol. 2014;5: 317 doi: 10.3389/fmicb.2014.00317 25071733 10.3389/fmicb.2014.00317 PMC4076661

[5] Díez-García M, Capita R, Alonso-Calleja C. Influence of serotype on the growth kinetics and the ability to form biofilms of *Salmonella* isolates from poultry. Food Microbiol. 2012;31(2): 173–180. doi: 10.1016/j.fm.2012.03.012 22608221 10.1016/j.fm.2012.03.012

[6] Wang H, Ding S, Wang G, Xu X, Zhou G. In situ characterization and analysis of *Salmonella* biofilm formation under meat processing environments using a combined microscopic and spectroscopic approach. Int J Food Microbiol. 2013;167(3): 293–302. doi: 10.1016/j.ijfoodmicro.2013.10.005 24184607 10.1016/j.ijfoodmicro.2013.10.005

[7] MacKenzie KD, Palmer MB, Köster WL, White AP. Examining the link between biofilm formation and the ability of pathogenic *Salmonella* strains to colonize multiple host species. Front Vet Sci. 2017;4: 138 doi: 10.3389/fvets.2017.00138 29159172 10.3389/fvets.2017.00138 PMC5581909

[8] Costerton JW, Stewart PS, Greenberg EP. Bacterial biofilms: a common cause of persistent infections. Science. 1999;284(5418): 1318–1322. 10334980 10.1126/science.284.5418.1318

[9] Zhang T, Fang HHP. Quantification of extracellular polymeric substances in biofilms by confocal laser scanning microscopy. Biotechnol. 2001;23(5): 405–409.

[10] Marshall JM, Flechtner AD, La Perle KM, Gunn JS. Visualization of extracellular matrix components within sectioned *Salmonella* biofilms on the surface of human gallstones. PLoS ONE. 2014;9(2): e89243 doi: 10.1371/journal.pone.0089243 24551241 10.1371/journal.pone.0089243 PMC3925243

[11] Buzón-Durán L, Alonso-Calleja C, Riesco-Peláez F, Capita R. Effect of sub-inhibitory concentrations of biocides on the architecture and viability of MRSA biofilms. Food Microbiol. 2017;65: 294–301. doi: 10.1016/j.fm.2017.01.003 28400016 10.1016/j.fm.2017.01.003

[12] Kirby AE, Garner K, Levin BR. The relative contributions of physical structure and cell density to the antibiotic susceptibility of bacteria in biofilms. Antimicrob Agents Chemother. 2012;56(6): 2967–2975. doi: 10.1128/AAC.06480-11 22450987 10.1128/AAC.06480-11 PMC3370779

[13] Sutherland I. Biofilm exopolysaccharides: a strong and sticky framework. Microbiol. 2001;147: 3–9. 10.1099/00221287-147-1-3 11160795

[14] Kamjunke N, Spohn U, Füting M, Wagner G, Scharf E, Sandrock S., et al Use of confocal laser scanning microscopy for biofilm investigation on paints under field conditions. Int Biodeterior Biodegradation. 2012;69: 17–22.

[15] Murga R, Stewart PS, Daly D. Quantitative analysis of biofilm thickness variability. Biotechnol Bioeng. 1995;45(6): 503–510. doi: 10.1002/bit.260450607 18623250 10.1002/bit.260450607

[16] Midelet G, Carpentier B. Impact of cleaning and disinfection agents on biofilm structure and on microbial transfer to a solid model food. J Appl Microbiol. 2004;97(2): 262–270. doi: 10.1111/j.1365-2672.2004.02296.x 15239692 10.1111/j.1365-2672.2004.02296.x

[17] Bridier A, Dubois-Brissonnet F, Boubetra A, Thomas V, Briandet R. The biofilm architecture of sixty opportunistic pathogens deciphered using a high throughput CLSM method. J Microbiol Methods. 2010;82(1): 64–70. doi: 10.1016/j.mimet.2010.04.006 20433880 10.1016/j.mimet.2010.04.006

[18] Chavant P, Martinie B, Meylheuc T, Bellon-Fontaine MN, Hebraud M. *Listeria monocytogenes* LO28: surface physicochemical properties and ability to form biofilms at different temperatures and growth phases. Appl Environ Microbiol. 2002;68(2): 728–737. doi: 10.1128/AEM.68.2.728-737.2002 11823213 10.1128/AEM.68.2.728-737.2002 PMC126664

[19] Petrova OE, Sauer K. Escaping the biofilm in more than one way: desorption, detachment or dispersion. Curr Opin Microbiol. 2016;30: 67–78. doi: 10.1016/j.mib.2016.01.004 26826978 10.1016/j.mib.2016.01.004 PMC4821722

[20] Flemming H-C, Wingender J. The biofilm matrix. Nat Rev Microbiol. 2010;8: 623–633. doi: 10.1038/nrmicro2415 20676145 10.1038/nrmicro2415

[21] Guilbaud M, Piveteau P, Desvaux M, Brisse S, Briandet R. Exploring the diversity of *Listeria monocytogenes* biofilm architecture by high-throughput confocal laser scanning microscopy and the predominance of the honeycomb-like morphotype. Appl Environ Microbiol. 2015;81(5): 1813–1819. doi: 10.1128/AEM.03173-14 25548046 10.1128/AEM.03173-14 PMC4325147

[22] Mulcahy H, Charron-Mazenod L, Lewenza S. Extracellular DNA chelates cations and induces antibiotic resistance in *Pseudomonas aeruginosa* biofilms. PLoS Pathog. 2008;4(11): e1000213 doi: 10.1371/journal.ppat.1000213 19023416 10.1371/journal.ppat.1000213 PMC2581603

[23] Mohammed MMA, Nerland AH, Al-Haroni M, Bakken V. Characterization of extracellular polymeric matrix, and treatment of *Fusobacterium nucleatum* and *Porphyromonas gingivalis* biofilms with DNase I and proteinase K. J Oral Microbiol. 2013;5: 20015. 10.3402/jom.v5i0.20015 PMC3559756 23372876

[24] Fish KE, Collins R, Green NH, Sharpe RL, Douterelo I, Osborn AM, et al Characterization of the physical composition and microbial community structure of biofilms within a model full-scale drinking water distribution system. PLoS ONE. 2015;10(2): e0115824 doi: 10.1371/journal.pone.0115824 25706303 10.1371/journal.pone.0115824 PMC4338064

[25] Simões M, Pereira MO, Vieira MJ. Effect of mechanical stress on biofilms challenged by different chemicals. Water Res. 2005;39(20): 5142–5152. doi: 10.1016/j.watres.2005.09.028 16289205 10.1016/j.watres.2005.09.028

[26] Xiao J, Koo H. Structural organization and dynamics of exopolysaccharide matrix and microcolonies formation by *Streptococcus mutans* in biofilms. J Appl Microbiol. 2010;108(6): 2103–2113. doi: 10.1111/j.1365-2672.2009.04616.x 19941630 10.1111/j.1365-2672.2009.04616.x

[27] Arciola CR, Campoccia D, Ravaioli S, Montanaro L. Polysaccharide intercellular adhesin in biofilm: structural and regulatory aspects. Front Cell Infect Microbiol. 2015;5: 7 doi: 10.3389/fcimb.2015.00007 25713785 10.3389/fcimb.2015.00007 PMC4322838

[28] Houari A, Seyer D, Kecili K, Heim V, Di Martino P. Kinetic development of biofilm on NF membranes at the Méry-sur-Oise plant, France. Biofouling. 2013;29(2): 109–118. doi: 10.1080/08927014.2012.752464 23320545 10.1080/08927014.2012.752464

[29] Möhle R, Langemann T, Haesner M, Augustin W, Scholl S, Neu TR, et al Structure and shear strength of microbial biofilms as determined with confocal laser scanning microscopy and fluid dynamic gauging using a novel rotating disc biofilm reactor. Biotechnol Bioeng. 2007;98(4): 747–755. doi: 10.1002/bit.21448 17421046 10.1002/bit.21448

[30] Wagner M, Ivleva NP, Haisch C, Niessner R, Horn H. Combined use of confocal laser scanning microscopy (CLSM) and Raman microscopy (RM): Investigations on EPS-Matrix. Water Res. 2009;43(1): 63–76. doi: 10.1016/j.watres.2008.10.034 19019406 10.1016/j.watres.2008.10.034

[31] Castelijn GA, van der Veen S, Zwietering MH, Moezelaar R, Abee T. Diversity in biofilm formation and production of curli fimbriae and cellulose of *Salmonella* Typhimurium strains of different origin in high and low nutrient medium. Biofouling. 2012;28(1): 51–63. doi: 10.1080/08927014.2011.648927 22235813 10.1080/08927014.2011.648927

